# Antimicrobial Activity and Chromatographic Analysis of Extracts from *Tropaeolum pentaphyllum* Lam. Tubers

**DOI:** 10.3390/molecules21050566

**Published:** 2016-04-28

**Authors:** Ritiel Corrêa da Cruz, Laura Bedin Denardi, Natalia Jank Mossmann, Mariana Piana, Sydney Hartz Alves, Marli Matiko Anraku de Campos

**Affiliations:** 1 Departamento de Farmácia Industrial, Universidade Federal de Santa Maria (UFSM), Avenida Roraima 1000, Block 26, Santa Maria 97105-900, Rio Grande do Sul, Brazil; natimossmann@gmail.com (N.J.M.); marianapiana@gmail.com (M.P.); 2 Departamento de Parasitologia, Microbiologia e Imunologia, Universidade Federal de Santa Maria (UFSM), Avenida Roraima 1000, Block 20, Santa Maria 97105-900, Rio Grande do Sul, Brazil; laura-denardi@hotmail.com (L.B.D.); sydneyalves.ufsm@gmail.com (S.H.A.); 3 Departamento de Análises Clínicas e Toxicológicas, Universidade Federal de Santa Maria (UFSM), Avenida Roraima 1000, Block 26, Santa Maria 97105-900, Rio Grande do Sul, Brazil; marlimatiko@yahoo.com

**Keywords:** *Tropaeolum pentaphyllum*, essential oil, benzyl isothiocyanate, antifungal, antibacterial

## Abstract

Background: *Tropaeolum pentaphyllum* Lam. tubers (Tropaeolaceae) are known and used as a condiment and for the treatment of skin infections in Southern Brazil. However, its activity and composition has not yet been investigated. Thus, different extracts and the essential oil from the tubers were tested against a range of microorganisms. The most active extracts were submitted to chromatographic analysis. Methods: Hydroalcoholic extract (70%), fractions of it, and the essential oil from the tubers were tested against several bacteria, yeasts and molds, furnishing the corresponding inhibitory, bactericidal and fungicidal minimal concentration values. The most active extracts were submitted to GC-MS investigation. Results: The strongest effects against different strains of microorganisms, such as Gram-positive and negative bacteria, *Candida* spp. and dermatophytes were observed for the essential oil and the chloroform fraction, with minimal inhibitory concentrations (MICs) well below 200 µg/mL. GC-MS analysis revealed that the major essential oil constituent is benzyl isothiocyanate (BITC), while the chloroform fraction is constituted of BITC, amides, sulfur, fatty acids and its esters, all compounds that may be related to the demonstrated activity. Conclusions: Overall, the results support the popular use of the plant for the treatment of skin infections, and revealed the main active compounds.

## 1. Introduction

*Tropaeolum pentaphyllum* Lam. (Tropaeolaceae) is a summer-growing species, native to South America, specifically Uruguay, Northern Argentina and Southern Brazil. It is used as an ornamental, food and medicinal plant. Its flowers make it known and appreciated throughout the world as an ornamental plant, but they are also locally consumed as part of salads [1] and used as an antidiabetic drug [2].

While the flowers of *T. pentaphyllum* have their applications, the most known and consumed part of the plant is its tubers. These can reach up to 1.5 kg in weight in due time, and are commonly known in south of Brazil as “crem” or “batata-crem” (crem-potato) and also “raiz-amarga” (bitter-root) [3]. “Crem” is a word derived from the slavic “chren”, a common denomination of the roots of *Armoracia rusticana* (horseradish) in Eastern Europe. *T. pentaphyllum*’s tubers and horseradish are commonly mistaken, as both are used as spices, prepared in the same way, as homemade pickles with red wine vinegar [4]. *T. pentaphyllum*’s tubers have similar organoleptic characteristics to those of the horseradish, and its consumption became popular during the 19th century with the settling of immigrants from Europe in the southern region of Brazil.

In traditional medicine, the consumption of the tubers is indicated to prevent and aid in the treatment of flu and scurvy. The decoction of the tubers is recommended as an option for the treatment of dermatosis and dermatological affections [5]. To the best of our knowledge, there is no scientific evidence corroborating this use, nor there are any studies demonstrating the presence of compounds related to these properties.

Bacterial and fungal infections represent part of the known and reported dermatological afflictions. Their treatments can be lengthy and expensive, requiring topical and parenteral medication, and as with other microbial infections, the microorganisms responsible can develop resistance mechanisms [6]. Therefore, the aim of the present study was to evaluate the popular use and the potential of *T. pentaphyllum* tubers, through an assessment of its *in vitro* antimicrobial activity and an investigation of the major constituents. For that, phytochemical analysis and antimicrobial evaluation against a series of microorganisms were conducted with the hydroalcoholic extract, its derived fractions and the essential oil obtained from the tubers.

## 2. Results

### 2.1. Extraction Methods

The dried tubers, extracted with hydroalcoholic solution (70%), yielded 20.2% of dried crude extract (CE). Yields for the fractions of the CE were 20.2% for the butanol fraction (BuF), 13.4% for the chloroform fraction (CfF) and 3.1% for the ethyl acetate fraction (EaF). The hydrodistillation extraction of the tubers yielded 0.082% of a limpid and pungent oil.

### 2.2. Antimicrobial Activity

Antimicrobial activity results are summarized in the Table 1, Table 2 and Table 3, divided among bacteria, yeasts and filamentous fungi. The CE and BuF did not show activity at the highest tested concentration (1280 µg/mL) against any of the tested microorganisms (data not shown). Potent bacteriostatic and bactericidal activity were observed for the essential oil (EO) and CfF (Table 1), against both Gram-positive and negative bacteria. Stronger results were those obtained against the Gram-negative *E. coli* and *S. pullorum*, with MICs in the same range of the tested reference antimicrobial agent (azithromycin). In addition, bactericidal activity was also verified, albeit at higher concentrations.

Antifungal activity is presented on Table 2 and Table 3, divided between yeasts and filamentous fungi. Strong activity was observed for the EO, benzyl isothiocyanate (BITC) standard and CfF, for both fungal forms. Fungicidal effect of these samples was observed for the majority of the tested fungi, the exceptions being some of the filamentous fungi. Fluconazole-resistant yeasts, such as *C. dubliniensis* and *C. glabrata*, were sensitive to both BITC and CfF, showing a lower MIC value, as well a fungicidal effect.

### 2.3. Chromatographic Analysis

**Figure 1:**
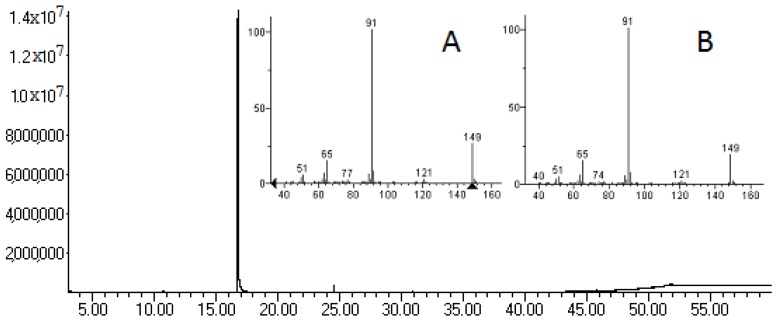
GC-MS chromatogram from the essential oil (EO) of *T. pentaphyllum* tubers, with mass spectra of benzyl isothiocyanate (BITC) standard from National Institute of Standards and Technology (NIST) library (**A**) and from peak with retention time of 16.76 min (**B**).

**Figure 2:**
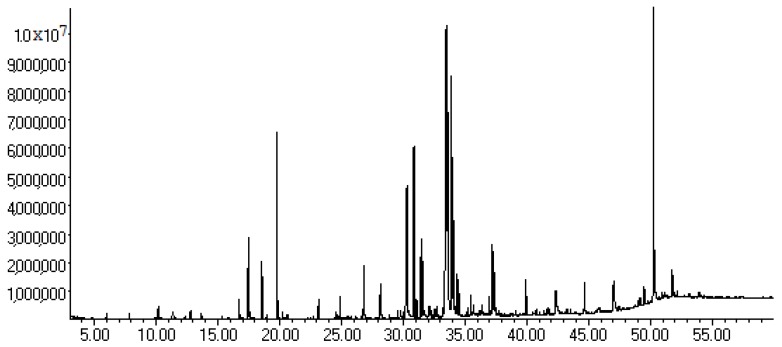
GC-MS chromatogram from the chloroform fraction of the CE from *T. pentaphyllum* tubers.

The most active tested samples in the antimicrobial activity evaluation were the EO and the CfF, as presented above, and therefore these were submitted to gas chromatography-mass spectrometry (GC-MS) analysis. The chromatograms are shown in Figure 1 and Figure 2. Table 4 complements Figure 2, presenting the composition of the CfF according to the peaks shown in the chromatogram.

As can be seen in Figure 1, the GC-MS analysis revealed that the major component of the oil is BITC. CfF is a more complex and varied extract, constituted by BITC, amides, sulfur, fatty acids and its esters, with some of the other major components described as a phytosterol and oleic acid (Table 4).

## 3. Discussion

Overall the antimicrobial activity was strong for the EO and the CfF, with MICs below the cutoff point for promising activity (*i.e.*, lower than 200 µg/mL) [7]. In addition to the bacteriostatic effect demonstrated by the low MIC values of the aforementioned extracts, bactericidal activity was also verified, albeit at higher concentrations. Since mostly of the current antibiotics are growth inhibitors, this bactericidal effect can be a relevant improvement, which also highlights a difference between mechanisms of action, indicating some direct action against the bacteria cell instead of protein synthesis inhibition mechanism (*i.e*., the mechanism of azithromycin) [8].

The antibacterial activity presented for the EO is due to its chemical composition. As presented in Figure 1, the GC-MS analysis revealed that the major component of the oil is BITC. While it is not a new compound to the genus, as it was already identified in *Tropaeolum majus* [9], this is the first report of its presence in *T. pentaphyllum*. The occurrence of BITC in *T. pentaphyllum* tubers explains their pronounced organoleptic properties, the reason behind its use as a condiment. 

The antibacterial activity of standard BITC was tested (Table 1), with results highly corresponding those of the EO. This is in accordance with previous publications, which demonstrated BITC bactericidal activity against a range of Gram-negative bacteria, while the tested Gram-positive bacteria were less susceptible [10]. Kim and Lee [11] demonstrated BITC activity against some harmful intestinal bacteria such as *Clostridium difficile* and *E. coli*, while not inhibiting other intestinal bacteria, such as *Bifidobacterium* spp. and *Lactobacillus acidophilus*, indicating that *T. pentaphyllum* tubers consumption could have a similar effect against intestinal bacteria. Dias *et al.* [12] showed that BITC is a stronger growth inhibitor against methicillin-resistant *Staphylococcus aureus* (MRSA) than allyl and 2-phenylethyl isothiocyanate, with MICs ranging from 2.9 to 110 µg/mL against several MRSA strains, the closest comparison that can be made with our results is that both BITC and EO had a 40 µg/mL MIC against a standard non-MRSA strain in our work.

Antimicrobial mechanisms of action of isothiocyanates (and BITC as well) are not well established, nonetheless several modes of action are proposed such as effects on membranes, inhibition of regulatory systems (quorum sensing), inhibition of respiratory enzymes, induction of heat-shock response, oxidative stress and stringent response [13]. Studies specifically with BITC demonstrated that it can cause the loss of membrane integrity, conversely to what was observed for ampicillin (a reference antibiotic) [10], and it was also the most potent inhibitor of the quorum sensing system CviIR on *Chromobacterium violaceum* when compared to allyl isothiocyanate and 2-phenylethyl isothiocyanate [14]. Other mechanisms of action verified with BITC were the induction of a heat-shock-like response, reduction of O_2_ consumption and protein aggregation on *Campylobacter jejuni* [15,16].

While the EO verified antibacterial activity is solely due to BITC, it is not the case for the CfF, which is a mixture of low polarity compounds as seen in Table 4. BITC is in fact part of this mixture, and it is followed in the chromatographic run by three structurally related compounds: 2-phenylacetamide, *N*-benzylacetamide and benzylcarbamide. To this moment it is not clear if these compounds are of natural occurrence, or derived from BITC, during the processing and extraction of the tubers. Similar amides were already found in plant extracts [17]. On the contrary, there is some evidence on isothiocyanate reaction products in aqueous medium, including the formation of elemental sulfur [18,19] a minor bacterial growth inhibitor [20], which may be related to the observed effect, since it was encountered in the CfF at 31.61 min.

Additionally contributing to the demonstrated antibacterial activity of CfF are fatty acids and their esters. They comprise the bulk of the fraction and possess antimicrobial activity [21]. Recently Tamokou *et al.* [22] purified a fraction from the ethyl acetate extract of *Albizia adianthifolias* stem bark containing only oleic and palmitic acid, two of the major constituents of the CfF, and it was active against *E. faecalis* and *S. aureus*, presenting MICs of 400 µg/mL and 200 µg/mL, respectively, which is less pronounced inhibitory activity than that exerted by CfF against the same bacteria (MICs of 80 and 40 µg/mL, respectively). In our study, we highlight the potent effect of the combined compounds of the fraction, rather than the action of individual constituents.

Not only was the antibacterial activity strong, but antifungal activity (Table 2 and Table 3) was also remarkable, observed for the EO, standard BITC and CfF, for both fungal forms. *T. rubrum* (Table 3), one of the major causes of dermatophytosis, was highly sensitive to both BITC and CfF, with a MIC value of 2.5 µg/mL, almost the same value obtained for the tested antifungal agent, itraconazole (MIC of 2.5 µg/mL). However, itraconazole is a fungistatic agent, whereas BITC and CfF also exerted a fungicidal effect, presenting MFCs of 40 µg/mL and 320 µg/mL, respectively. Another common causative agent of dermatophytosis, *M. canis*, was also very sensitive to BITC and CfF (MICs of 20 µg/mL). These data corroborates the widespread use of the tubers’ decoction to treat dermatophytosis [5]. Other filamentous fungi that causes cutaneous and subcutaneous infections, such as *F. pedrosoi* (chromoblastomycosis causative agent) and *S. schenckii* (sporotrichosis causative agent) were also sensitive to both BITC and CfF, while *Aspergillus* spp. presented mixed results, *A. flavus* and *A. niger* clinical isolates were resistant to CfF (MIC of 1280 µg/mL and with no observed fungicidal activity at the same concentration).

Antifungal activity of isothiocyanates against few fungi species was reported by Drobnica *et al.* [23] who showed that BITC is in general 3.6 time more potent than allyl isothiocyanate. Equivalent results can be seen, as the authors obtained a MIC of 26.86 µg/mL against filamentous fungus *A. niger* after four days of incubation, while we observed a MIC of 160 µg/mL for our clinical isolate of *A. niger*, with two days of incubation. More recently Manici *et al.* [24] showed isothiocyanates have fungitoxic activity against plant pathogenic fungi, and the proposed mechanisms of action were inactivation of intracellular enzymes by breakdown of disulfide bonds, inhibition of metabolic enzymes by thiocyanate radical (indicated as degradation product of isothiocyanates) and uncoupler action on oxidative phosphorylation.

While the antifungal activity of BITC is more established and explains the EO antifungal properties, the CfF presents a different situation, where activity can be ascribed to the whole set of compounds, instead of selected ones. Elemental sulfur (RT of 31.61 min), at 1.62% of the CfF, can play a major role in the observed antifungal activity. In fact, it is regarded as the oldest of all pesticides, with well-established antifungal action, whether in its inorganic or organic forms [25].

There are no reports in the literature regarding the activity of the amides of the CfF composition (eluted between 17.49 min and 19.80 min). However there are some indications that they can present some inhibitory properties. Antimicrobial activity of the ethyl acetate extract from the algae *Trichodesmium* *erythraeum* [26], containing, among other compounds, 2-phenylacetamide (benezeneacetamide) at 17.48%, inhibited the growth of some fungi such as *T. rubrum* and *Trichophyton simii*, with MICs of 500 µg/mL and 16.2 µg/mL. CfF was stronger against *T. rubrum*, with a MIC of 2.5 µg/mL, and 4.37% of 2-phenylacetamide in its composition, suggesting that these amides do not play a prominent role in the antifungal activity of the CfF, albeit a synergistic contribution is possible.

In addition to the BITC and elemental sulfur contribution to the observed antifungal activity, the fatty acids and its esters, which comprise the bulk of the CfF, may also have an important role. Extracts and fractions containing fatty acids have been reported to possess at least fungistatic activity. The results of Tamokou *et al.* [22] for a purified mixture of oleic and palmitic acid obtained from ethyl acetate extract of *Albizia adianthifolia* were active against different *Candida* sp. and *C. neoformans*, with MICs ranging from 100 µg/mL to 400 µg/mL, higher values than those observed for CfF against a wide range of tested yeasts.

Palmitic and oleic acid, major constituents of CfF (6.75% and 10.25%, respectively), are among the fatty acids reviewed for their antifungal properties [27]. A proposed possible mechanism of antifungal action of fatty acids indicates that they can alter fungal membrane fluidity, causing cell membrane disorganization and leakage of vital components, eventually leading to cell disintegration [28].

The importance of fatty acids in the plant, and in the extract, may not be restricted only to the antimicrobial activity, but also for their action as penetration enhancers, a supporting role in the utilization of the plant against skin infections. Fatty acids and their esters can penetrate the skin, but in addition they could also promote the penetration of other active compounds such as the hydrophobic BITC, the amides and sulfur, to deeper layers of the skin, by disruption and alteration of the stratum corneum lipid strucure [29,30]. An extract from the algae *Botryococcus braunii* rich in palmitic and oleic acid [31], fatty acids we identified in the CfF, enhanced the skin penetration of flurbiprofen, and while the extract were less effective than the purified fatty acids, it was also less irritating to the skin. Another work [32] demonstrated that fatty acids can penetrate and accumulate to different degrees in the skin, and, in addition, the authors have shown that oleic acid significantly enhanced tolnaftate penetration.

To the moment, of the compounds encountered in the EO and CfF of the tubers from *T. pentaphyllum*, only the fatty acids have an established relation with the skin. We have not found specific studies concerning BITC, amides and elemental sulfur. Their hydrophobic, and low molecular weight structures put them among candidates for skin penetration and enhancement by combined use along with fatty acids, although experimentation with these compounds is necessary to a better understanding of the behavior towards skin.

## 4. Materials and Methods

### 4.1. Plant Material and Extraction Methods

Tubers of *T. pentaphyllum* were acquired from local farmers in the municipality of Gaurama (Rio Grande do Sul, Brazil), in August 2011, along with the material required for identification. A dried voucher specimen, containing leaves and flowers, is preserved in the herbarium of the Biology Department at the Federal University of Santa Maria under the registration code SMDB*-*13137.

About 1.9 kg of fresh tubers were initially shredded, after that an amount of 200 g was separated for essential oil extraction and the remaining material was submitted to drying, at 40 °C, in an oven with air flow, during seven days. These shredded and dried tubers were then ground in a knife mill. The resulting dried tuber powder (517 g) was submitted to maceration, at room temperature during a week, with 70% ethanol in water, with a proportion of 8 mL of solvent for each g of plant material. The obtained crude extract (CE) was filtered and the alcoholic solution was evaporated under reduced pressure, in order to eliminate the ethanol. The remaining aqueous solution was successively partitioned with chloroform, ethyl acetate and *n*-butanol until depletion of the color visible components, which was achieved extracting 300 mL of the aqueous crude extract three times with 300 mL of chloroform, followed by three extractions with 300 mL of ethyl acetate and three extractions with butanol, using 300 mL, 200 mL and 150 mL of solvent. Each organic extract was subsequently dried under reduced pressure, resulting in chloroform (CfF), ethyl acetate (EaF) and *n*-butanol (BuF) dried fractions from the CE. In addition, an aliquot of the referred crude extract was fully dried, and stored for further use.

Essential oil (EO) from the tubers was obtained from the shredded fresh material (200 g), separated as mentioned above. Extraction was performed through hydrodistillation at 60 °C during four hours, using a Clevenger apparatus. Separation of the oil from the water was achieved in a separatory funnel.

### 4.2. Antimicrobial Assays

The essential oil, the hydroalcoholic extract and its fractions, as well as standard benzyl isothiocyanate (BITC) were tested against bacteria, yeasts and molds. Susceptibility tests were performed according to the Clinical and Laboratory Standards Institute (CLSI) microdilution technique, M07-A9 [33] for bacteria, M27-A3 [34] for yeasts and M38-A2 [35] for molds. Compounds were solubilized in dimethyl sulfoxide (DMSO) to obtain stock solutions with final concentration of 128,000 µg/mL. The solutions were used immediately after preparation, and all the analysis were executed in triplicate. Final concentration of DMSO in the testing wells was of 0.01%, experimentally determined to not interfere with the analysis. The tested concentrations ranged from 2.5 µg/mL to 1280 µg/mL for all of the microorganisms. The results of the tests were expressed as the minimal inhibitory concentration (MIC), minimal fungicidal concentration (MFC) and minimal bactericidal concentration (MBC).

#### 4.2.1. Antibacterial Activity

The following six bacteria strains were tested: *Enterococcus faecalis* ATCC 91299, *Escherichia coli* ATCC 5922, *Klebsiela pneumoniae* ATCC 700603, *Pseudomonas aeruginosa* ATCC 27853, *Salmonella pullorum* ATCC 9140, *Staphylococcus aureus* ATCC 29213. The isolates were stored in 10% glycerol solution at −70 °C and revived by subculturing in Mueller-Hinton Agar (MHA). The compounds were tested by microdilution broth assay using Mueller-Hinton Broth (MHB) medium in 96-well microplates. The inoculum was prepared by growing bacteria at 37 °C for 24 h in MHA. Colonial growth was suspended in 2 mL of saline, to an approximate 0.5 McFarland turbidity or 1 × 10^8^ colony-forming unit per mL (CFU/mL). The inoculum was diluted (1:20) in MHB and added to all wells, except negative control. The plates were incubated at 37 °C during 24 h. Antibacterial activity was detected by adding 20 µL of 0.5% TTC (triphenyltetrazolium chloride, Merck, Darmstadt, Germany) solution. MIC was defined as the lowest concentration of compounds that inhibited visible growth, as indicated by the TTC staining. Bacterial suspensions from all tests wells were subcultured in sterile agar medium in order to evaluate bactericidal activity.

#### 4.2.2. Antifungal Activity

Dilutions of the compounds were prepared in RPMI-MOPS 1640 medium, in order to obtain two times the final concentrations, and 100 µL of each concentration of extracts was added to columns 1 to 10 of each 96-well microplates. After, 100 µL of standard inoculum was added to all wells, except negative control. The plates were incubated for 48 h at 35 °C.

Strains of fourteen species of yeasts were used, including clinical isolates, caspofungin/fluconazole sensitive or resistant species and standard ATCC/CBS strains. The clinical isolates were obtained from the private collection of the Mycology Research Laboratory, Federal University of Santa Maria, Santa Maria, Brazil (LAPEMI-UFSM) and identified based on carbohydrate assimilation profiles using by ID32-C test (bioMérieux). The strains were grown in Sabouraud Dextrose Agar (SDA) for 48 h at 35 °C to inoculum preparation. The following yeast were used (*Candida albicans* ATCC 140053, *Candida dubliniensis* CBS 7987, *Candida dubliniensis* fluconazole-sensitive, *Candida dubliniensis* fluconazole-resistant, *Candida glabrata* ATCC 2001, *Candida glabrata* fluconazole-sensitive, *Candida glabrata* fluconazole-resistant, *Candida glabrata* caspofungin-resistant, *Candida guilliermondii*, *Candida parapsilosis* ATCC 22018, *Candida parapsilosis* caspofungin-resistant, *Candida tropicalis*, *Cryptococcus neoformans* ATCC 90012, *Saccharomyces cerevisiae* ATCC 260).

Strains of ten species of molds were used, including clinical isolates (CI) and environmental isolates (EI) as follows (*Aspergillus fumigatus* (CI), *Aspergillus fumigatus* (EI-isolated from maize), *Aspergillus flavus* (CI), *Aspergillus niger* (CI), *Trichophyton rubrum* (CI), *Microsporum canis* (CI), *Fonsecaea pedrosoi* (CI), *Pseudallescheria boydii* (CI), *Fusarium solani* (CI), *Sporothrix schenckii* (CI)). The clinical and environmental isolates were obtained from the private collection of the Mycology Research Laboratory, Federal University of Santa Maria, Santa Maria, Brazil (LAPEMI-UFSM) and identified by macroscopic and microscopic examination. The strains were grown in Potato Dextrose Agar (PDA) from 48 h until seven days at 35 °C for inoculum preparation.

The MIC is defined as the lowest concentration of the compound that inhibits the visible growth of a microorganism after 48 h of incubation, as indicated by the TTC staining. Fungal suspensions from all tests wells were subcultured in sterile Sabouraud Dextrose Agar medium in order to evaluate fungicidal activity.

### 4.3. Chromatographical Analysis

The essential oil and the chloroform fraction of the crude extract were submitted to separation through gas chromatography, with detection by mass spectrometry (GC-MS). The ethyl acetate and *n*-butanol fractions were not suitable for this analysis due to their physical and chemical properties, moreover, chloroform fraction exhibited better results in the antimicrobial activity assay making it the logical choice for a more in-depth investigation.

The analysis was performed in a model 6890N chromatograph, coupled with a 5975B model mass detector, both from Agilent Technologies. Chromatographic conditions were as follows: oven initial temperature was 50 °C, during one minute, followed by heating at a rate of 5 °C/min until 300 °C, remaining this temperature during 9 min, of a total of 60 min run; separation was achieved in a DB-5MS column (30 m × 320 µm × 0.25 µm), at a constant rate of 1.5 mL/min of helium; detection was performed in the quadrupole equipment using electron ionization. Peak identification was carried by comparison of the experimental mass spectrum with those of the National Institute of Standards and Technology (NIST) standard library and published papers.

## 5. Conclusions

The *in vitro* antimicrobial activity tested and reported corroborates the popular use of the plant’s tubers against cutaneous infections, signaling a possible alternative for the treatment of resistant skin infections, especially of fungal origin. The chemical findings suggest that more than one compound may be related to this effect, with the pro-eminence of benzyl isothiocyanate. Its presence not only explains and is related to the antimicrobial activity, but it is also relevant to its edible aspects. The reported results should pave the way for more investigations, aiming at determining the *in vivo* toxicity and pharmacological effects, and a more in-depth evaluation of the plant composition.

## Figures and Tables

**Table 1:** Antibacterial activity of the essential oil and fractions from the crude extract (CE) of *T. pentaphyllum* tubers.

Bacteria	MIC/MBC (µg/mL)				
	EO	CfF	EaF	BITC	Azithromycin
*Enterococcus faecalis* ATCC 91299	40/640	80/640	80/1280	40/640	8/-
*Escherichia coli* ATCC 5922	10/320	10/80	20/640	10/320	8/-
*Klebsiella pneumoniae* ATCC 700603	40/1280	40/640	320/-	40/640	2/-
*Pseudomonas aeruginosa* ATCC 27853	20/320	40/640	40/640	20/320	4/-
*Salmonella pullorum* ATCC 9140	40/640	10/-	80/1280	40/640	8/-
*Staphylococcus aureus* ATCC 29213	40/1280	40/640	640/-	40/320	2/-

EO: Essential oil; CfF: Chloroform fraction; EaF: Ethyl acetate fraction; BITC: Benzyl isothiocyanate; -: Not active.

**Table 2:** Antifungal activity of the essential oil and fractions from the CE of *Tropaeolum* *pentaphyllum* tubers, against yeast fungi.

Fungus	MIC/MFC (µg/mL)				
	EO	CfF	EaF	BITC	Fluconazole
*Candida albicans* ATCC 14053	40/320	20/20	40/320	40/160	4/-
*Candida dubliniensis* CBS7987	40/320	10/10	40/640	40/160	8/-
*Candida dubliniensis* CI FS	40/160	20/20	40/640	40/160	2/-
*Candida dubliniensis* CI FR	40/160	20/20	40/640	40/160	64/-
*Candida glabrata* ATCC 2001	20/80	40/40	40/320	20/80	32/-
*Candida glabrata* CI FS	20/160	40/40	40/640	20/160	16/-
*Candida glabrata* CI FR	20/160	40/40	20/640	20/160	128/-
*Candida glabrata* CI CR	NT	160/1280	NT	160/640	4/-
*Candida guilliermondii* CI	80/320	10/10	80/320	80/320	8/-
*Candida parapsilosis* ATCC 22018	80/320	10/20	80/640	80/320	16/-
*Candida parapsilosis* CI CR	NT	320/1280	NT	320/1280	8/-
*Candida tropicalis* CI	320/-	10/10	1280/-	320/-	8/-
*Cryptococcus neoformans* ATCC 90012	10/80	2.5/2.5	10/320	5/80	16/-
*Sacharomyces cerevisiae* ATCC 2601	40/320	2.5/2.5	80/320	40/160	4/-

EO: Essential oil; CfF: Chloroform fraction; EaF: Ethyl acetate fraction; BITC: Benzyl isothiocyanate; -: Not active; NT: Not tested; CI: Clinical isolate; FS: Fluconazole sensitive; FR: Fluconazole resistant; CR: Caspofungin resistant.

**Table 3:** Antifungal activity of the essential oil and fractions from the CE of *T. pentaphyllum* tubers, against dermatophytes and filamentous fungi.

Fungus	MIC/MFC (µg/mL)				
	EO	CfF	EaF	BITC	Itraconazole
*Aspergillus fumigatus* CI	80/1280	160/1280	320/-	80/1280	8/-
*Aspergillus fumigatus* EI	20/1280	80/640	40/-	20/1280	16/-
*Aspergillus flavus* CI	NT	1280/-	NT	160/1280	1/-
*Aspergillus niger* CI	NT	1280/-	NT	160/1280	2/-
*Trichophyton rubrum* CI	NT	2.5/320	NT	2.5/40	2/-
*Microsporum canis* CI	NT	20/160	NF	20/80	8-
*Fonsecaea pedrosoi* CI	NT	80/640	NT	40/320	1/-
*Pseudallescheria boydii* CI	NT	80/1280	NT	40/640	4/-
*Fusarium solani* CI	NT	320/-	NT	160/1280	>16/-
*Sporothrix schenckii* CI	NT	40/320	NT	80/320	4/-

EO: Essential oil; CfF: Chloroform fraction; EaF: Ethyl acetate fraction; BITC: Benzyl isothiocyanate;-: Not active; NT: Not tested; CI: Clinical isolate; EI: Environmental isolate; FS: Fluconazole sensitive; FR: Fluconazole resistant; CR: Caspofungin resistant.

**Table 4:** Identified compounds from chloroform fraction (CfF) of the CE from *T. pentaphyllum* tubers by GC-MS.

Compound	RT (min)	Relative Area (%)	(M^+^), *m*/*z* (%)	Major Fragment Ions, *m*/*z* (%)
Benzyl isothiocyanate	16.72	0.65%	149 (16)	91 (100) 65 (14) 92 (7) 89 (5) 63 (5) 51 (4) 50 (3) 90 (3) 62 (2)
2-Phenylacetamide	17.49	4.37%	135 (18)	91 (100) 92 (91) 65 (20) 44 (12) 63 (8) 89 (7) 51 (5) 93 (5) 90 (4)
*N*-benzylacetamide	18.56	2.21%	149 (71)	106 (100) 91 (28) 43 (20) 107 (15) 77 (13) 79 (13) 51 (8) 65 (7) 150 (7)
Benzylcarbamide	19.80	6.23%	150 (50)	106 (100) 79 (41) 91 (34) 77 (34) 51 (23) 107 (20) 78 (15) 104 (15) 65 (13)
Palmitic acid	30.29	6.75%	256 (30)	73 (100) 60 (84) 43 (73) 57 (63) 55 (57) 41 (56) 129 (43) 71 (37) 69 (31) 83 (24)
Ethyl palmitate	30.86	4.66%	284 (7)	88 (100) 101 (58) 43 (26) 55 (22) 41 (20) 89 (17) 57 (16) 70 (16) 157 (15) 73 (15)
Elemental sulfur	31.61	1.62%	256 (70)	64 (100) 160 (53) 128 (52) 192 (41) 258 (25) 32 (21) 96 (21) 224 (18) 66 (11)
Oleic acid	33.60	10.25%	282 (1)	55 (100) 69 (72) 41 (67) 83 (58) 97 (49) 67 (45) 43 (45) 81 (37) 84 (34) 57 (32)
Ethyl-9,12-octadecadienoate	33.90	6.98%	308 (8)	67 (100) 81 (90) 95 (65) 55 (61) 41 (46) 79 (43) 82 (42) 68 (38) 96 (35) 54 (34)
Ethyl oleate	34.02	5.88%	310 (6)	55 (100) 69 (71) 41 (63) 83 (60) 88 (57) 97 (51) 84 (49) 43 (47) 96 (45) 101 (45)
Butyl palmitate	34.38	1.03%	312 (6)	56 (100) 57 (47) 257 (41) 43 (35) 41 (32) 55 (28) 73 (24) 60 (24) 239 (20) 129 (20)
Unknown phytosterol	50.26	11.35%	414 (100)	43(95) 107 (68) 105 (66) 145 (65) 95 (62) 55 (61) 81 (60) 329 (59) 303 (58)

RT: retention time.
